# Management of the Sickle Cell Trait: An Opinion by Expert Panel Members

**DOI:** 10.3390/jcm12103441

**Published:** 2023-05-12

**Authors:** Valeria Maria Pinto, Lucia De Franceschi, Barbara Gianesin, Antonia Gigante, Giovanna Graziadei, Letizia Lombardini, Giovanni Palazzi, Alessandra Quota, Rodolfo Russo, Laura Sainati, Donatella Venturelli, Gian Luca Forni, Raffaella Origa

**Affiliations:** 1 Centro della Microcitemia, Anemie Congenite e Dismetabolismo del Ferro, E.O. Ospedali Galliera, 16128 Genova, Italy; barbara.gianesin@galliera.it (B.G.); gianluca.forni@galliera.it (G.L.F.); 2 Dipartimento di Medicina AOUI, Università di Verona, 37124 Verona, Italy; lucia.defranceschi@univr.it; 3 ForAnemia Foundation, 16124 Genova, Italy; antonia.gigante@foranemia.org; 4 Società Italiana Talassemie ed Emoglobinopatie (SITE), 09100 Cagliari, Italy; 5 Centro Malattie Rare Internistiche, Medicina Generale, Fondazione IRCCS Ca’ Granda Ospedale Maggiore Policlinico, 20122 Milano, Italy; giovanna.graziadei@policlinico.mi.it; 6 Centro Nazionale Trapianti, Istituto Superiore di Sanità, 00161 Roma, Italy; letizia.lombardini@iss.it; 7 U.O. Oncoematologia Pediatrica, Azienda Ospedaliero-Universitaria di Modena, 41125 Modena, Italy; palazzi.giovanni@policlinico.mo.it; 8 UOSD Talassemia, P.O. Vittorio Emanuele, 93012 Gela, Italy; alequota@hotmail.com; 9 Clinica Nefrologica, Dialisi e Trapianto, Dipartimento di Medicina Integrata con il Territorio, IRCCS Ospedale Policlinico San Martino, 16132 Genova, Italy; rodolfo.russo@hsanmartino.it; 10 Oncoematologia Pediatrica, Azienda Ospedaliera-Università di Padova, 35128 Padova, Italy; sainatilaura@gmail.com; 11 Servizio Immunotrasfusionale, Azienda Ospedaliero-Universitaria di Modena, 41125 Modena, Italy; venturelli.donatella@aou.mo.it; 12 Talassemia, Ospedale Pediatrico Microcitemico ‘A.Cao’, ASL8, Università di Cagliari, 09121 Cagliari, Italy

**Keywords:** sickle cell trait, hemoglobinopathies, exertional rhabdomyolysis, pregnancy, kidney disease, pain, spleen infarction, surgery, transplantation, specialist advice

## Abstract

The number of individuals with the sickle cell trait exceeds 300 million worldwide, making sickle cell disease one of the most common monogenetic diseases globally. Because of the high frequency of sickle cell disease, reproductive counseling is of crucial importance. In addition, unlike other carrier states, Sickle Cell Trait (SCT) seems to be a risk factor for several clinical complications, such as extreme exertional injury, chronic kidney disease, and complications during pregnancy and surgery. This expert panel believes that increasing knowledge about these clinical manifestations and their prevention and management can be a useful tool for all healthcare providers involved in this issue.

## 1. Introduction

The sickle cell trait is the carrier state for the sickle hemoglobin (HbS) mutation. HbS is the result of a point mutation on a single nucleotide base of the gene encoding the beta (β) subunit of hemoglobin chains. As a result of this mutation, an adenine replaces a thymine, causing the replacement of one amino acid (glutamic acid) with another (valine) [1]. In conditions of low oxygen tension, abnormal HbS polymerizes and erythrocytes stretch and bend, assuming the characteristic sickle shape; they can build up, producing obstructions to circulation that, especially in its homozygous form (drepanocytosis, sickle cell anemia or sickle cell disease (SCD)), can cause painful health emergencies, such as vaso-occlusive crises and other possibly serious consequences. It affects both quality of life and life expectancy [2,3,4,5,6].

The sickle cell trait (SCT) condition is frequently found in patients of African origin or in those who come from regions where malaria is endemic (such as tropical and subtropical regions). Approximately 300 million people are thought to be affected by SCT worldwide, approximately one-third of whom live in subSaharan Africa [7,8,9,10]. The prevalence of SCT in some of these areas is over 25% and can even reach 45% (Nigeria [11]). As a result of the slave trade and, more recently, of migration flows, the HbS gene has spread in America, Europe, and Australia. The prevalence of SCT in the United States of America affects 7–9% of the African American population (equal to approximately 3 million people) and 0.2% of the Caucasian population. In Italy, HbS was, historically, almost exclusively present in southern regions, especially in Sicily, where SCT prevalence reaches peaks of 13% in some areas. However, as a result of the south–north migration flows and of more recent international migration flows (from, for example, Africa and Albania), SCT is now present throughout the Italian national territory [12,13].

The association between malaria and SCT was reported for the first time by Allison in 1954 [14]. Although patients affected by SCT are not protected from parasitemia when exposed to Plasmodium falciparum, they are nevertheless unlikely to develop severe consequences from malarial infection, with a reduction of approximately 90% in the infection’s clinical impact [15].

HbS concentration can vary considerably from 25 to 45%; it generally stands at approximately 40%. In the African American population, the co-presence of a deletion of the alpha-globin gene (−α/αα, present in 30% of the population) or of two alpha-globin genes (−α/−α, present in 2% of the population) can cause a variation in HbS equal to approx. 37% and 29%, respectively [16].

A person with SCT status usually has a normal life expectancy and quality of life [8]. However, very rarely and under certain metabolic or environmental conditions, such as hypoxia, acidosis, dehydration, hyperosmolarity, and hyperthermia, some complications of the sickle cell disease can occur. Therefore, specific precautions must be taken to avoid possible dehydration conditions in the event of prolonged exposure to heat, heatstroke, or low oxygen tension, as could occur in high mountains or severe physical stresses, such as those experienced in certain types of sports activities and during major surgeries.

Moreover, the association between SCT and red blood cell disease due to the presence of membrane or enzymatic defects (spherocytosis and stomatocytosis) could exacerbate the phenotypic expression of SCT. Therefore, especially in “symptomatic” cases of SCT, the coexistence of possible red blood cell membrane or enzymatic defects should be investigated.

Hence, although SCT is largely considered to be a benign condition, it may in fact be a risk factor for some clinical symptoms.

The aim of this work is to outline the extent to which and under what conditions SCT may constitute a risk factor, providing information and outlining preventive measures to avoid the occurrence of such conditions.

This paper is divided into topics with specific questions answered by experts who report the available evidence and formulate relevant recommendations. Ten areas of interest were identified from which questions may arise. The identified areas are the communication of SCT status, sports activities, kidney disease, stroke, thrombophilia, bone disease, transplant and donation, neonatal/pediatric age, pregnancy, and eye disease. A miscellaneous section (Section 12) is also presented in which we address specific questions related to the risk of splenic infarction, infections, vaso-occlusive crises, and surgeries. Medline, PubMed, Embase, and the Cochrane Library were searched for bibliographies, using the following keywords: sickle cell trait; hemoglobinopathies; pregnancy; exercise; sudden death; transplantation; genetic counselling; osteonecrosis; stroke; kidney disease; infections; pain; spleen infarction; surgery.

The major clinical complications of SCT and proposed counseling recommendations are summarized in Table 1.

## 2. SCT and Communication

### 2.1. Should the Family Be Informed of the SCT Carrier Status Once It Is Identified?

The identification of the carrier status is a consequence of screening programs or of the tests taken in the event of suspected sickle cell disease or other hemoglobinopathies. The decision to inform the patient or his/her parents of their carrier status is not univocal, due to the possible implications related to stigmatization or work or insurance limitations the carrier might face.

Some screening programs do not allow the communication of the carrier status. In Germany, a national law, the German Genetic Testing Act (GenDG) [17,18], has regulated the acquisition and dissemination of genetic inheritance information since 2010; therefore, SCT cases are not even registered [19]. In other nations, the discussion regarding the option to inform patients about their carrier status remains open because of the risk of overmedicalizing a fundamentally healthy individual or “vulnerable child” [20].

In the United States, there are no national standards regulating the notification and registration of the carrier; each state has its own protocol with wide procedural variability. In addition to this, in most cases, it is the treating physician who is informed and not the parents, thus increasing the risk of the patient not being informed [21]. Some argue that this partially impedes the effectiveness of newborn screening (NBS) programs, which can almost be considered missed opportunities, because detailed information about the carrier status and the consequent implications should be provided at least three moments during a patient’s life: the neonatal period, to assess the implications for any subsequent pregnancies; preadolescence, for the prevention of any risks related to sporting activities; and late adolescence–young adulthood, for the implications related to reproductive risk [22].

In some Italian regions, the screening programs that began in the 1970s have increased awareness about thalassemia and achieved the goal of preventing it, as well as decreasing the number of children born with SCD [23,24,25].

This expert panel believes it is important to identify and communicate the SCT carrier status, above all for the positive consequences that this information might have both for patients’ awareness of the reproductive risk and for the health of the subject.

### 2.2. Is Specialist Advice Useful for SCT Carriers?

Preconceptional counselling of at-risk couples (SCD and SCT) has been unsuccessful in many contexts as far as the control of reproductive risk is concerned, even when accompanied by information campaigns [26]. A recent prospective randomized trial seems to demonstrate that awareness of reproductive risks is more successfully gained when the information and the educational pathway are specific and communicated by competent personnel, who are experts in the specific disease [27]. However, there are no studies with observation periods long enough to evaluate the effects of specialist advice on young people and on more mature adults. An information/prevention campaign in the Italian territory, i.e., in an advanced social and healthcare context, might produce better results, as has already happened for thalassemia [25].

Specialist advice, communicated by specially trained staff who are experts in sickle cell disease, is recommended for all SCT carriers during adolescence.

## 3. SCT and Sporting Activities

All preventive measures described here in relation to the possible effects of intense physical exertion should be taken by all athletes in order to safeguard their health.

### 3.1. Is There an Increased Risk of Exercise-Related Morbidity and/or Mortality in the SCT Population?

Exercise-related morbidity and mortality in individuals affected by SCT are mainly linked to a very rare clinical issue referred to as exertional rhabdomyolysis and to exercise-related sudden death, which can occur under extreme conditions, such as severe dehydration and high-intensity physical activity.

Studies report that severe or fatal exertional rhabdomyolysis is attributable to a wide range of activities (football, training, cross-country racing, swimming, spinning, hockey, exercises in the army, etc.) in which the effort undergone by the individual can be classified as intense. The same studies also describe compartment syndromes, hematuria and isosthenuria [28,29].

During intense physical exertion, exertional rhabdomyolysis manifests as extreme muscle weakness and pain, even mild pain, which arises rapidly without prodromes. Rhabdomyolysis can be fulminant with muscle cell death, potassium release, hypercalcemia, and the death of the subject due to the fact of arrythmia [30,31].

Documented sports activities, the clinical picture and evolution, the causes of exertional sickling, and inducing and protective factors are summarized in Table 2.

Several studies conducted in a military environment and, above all in, football [32] report that sudden death is the most dangerous complication in individuals, athletes, and soldiers with SCT [33,34]. The risk of sudden death in recruits with SCT during intense exertion (assessed in 2 million military recruits in the USA) is estimated to be 30 times higher among African American individuals and 40 times higher than that in recruits in general [35]. Death is mainly attributed to exertional rhabdomyolysis during the first month of training and during activities requiring high-intensity exercises [36]. The increase in deaths correlates with age (higher at 28–29 years than at 17–18 years), probably due to the cumulative effect of renal papillary necrosis and the resulting isosthenuria, which is common in SCT (it is observed in 85% of individuals of the age of male recruits [35]). In more recent studies, HbAS was associated with a higher adjusted risk of exertional rhabdomyolysis for military men (hazard ratio: 1.54; 95% CI 1.12 to 2.12; *p* = 0.008). The risk was 57% higher in men >36 years old [37].

### 3.2. What Precautions Should Be Taken?

If preventive measures are taken, the risk of sudden death attributed to SCT seems to be zeroed [37].

Hydration and training with progressive exercises can “revert”/normalize some hematological abnormalities (increased plasma viscosity, oxidative stress, endothelial activation, and sickling) observed during exertion [32].

Coaches, athletic trainers, and medical staff should follow universal training precautions [38]. Coaches must be aware of the potential dangers during training; therefore, they should avoid repetitive and intense activities without breaks and stop the exercises immediately and alert the doctor if an athlete shows signs of exertional rhabdomyolysis.

It is essential to prepare an emergency management plan in advance, as well as ensuring that the necessary equipment is available during all training sessions and competitions.

### 3.3. Can Individuals with SCT Practice Sports Activities and Undertake Them at a Competitive Level?

The benefits of (aerobic and anaerobic) physical activity in SCT carriers are similar to those experienced by individuals without SCT; therefore, SCT carriers should be encouraged to practice sports.

However, sports clubs and coaches must implement strategies to reduce any risks associated with the player’s SCT status; these include appropriate training and monitoring during training.

If the athlete is aware of his/her SCT status, appropriate precautions should be taken regarding individualized training methods and resting periods.

If the SCT status is not known, preventive measures should be adopted anyway to safeguard all athletes.

It is important that coaches and physical trainers are aware of the possible presence of SCT carriers among their athletes and that training sessions in general include appropriate rest intervals and hydration. In addition to this, coaches and trainers should be able to recognize the early signs of specific symptoms that may arise during physical exertion in athletes with SCT and to initiate early treatment.

No contraindications to sports activities are reported for athletes with SCT.

### 3.4. Can Individuals with SCT Practice High-Altitude (e.g., Mountain Climbing) or High-Pressure (e.g., Scuba Diving) Activities?

(A) High-altitude activities

Only individual cases are reported in the literature; there are no studies with large numbers of splenic infarctions secondary to high-altitude flights in unpressurized aircraft or during mountaineering excursions. Usually, the altitude at which athletes can train or compete is between 2000 and 2500 m, and this is defined as a moderate altitude.

Intense physical activity at low altitudes is certainly a risk factor for rare complications, such as splenic infarction, sudden death, or rhabdomyolysis; rapid exposure to high altitudes and related hypoxia increase the frequency of splenic infarction episodes. In retrospective studies, over 90% of the cases of patients with splenic infarctions at high altitude were SCT and males.

In this context, hypoxia and physical stress are responsible for vaso-occlusion from sickling in the spleen red pulp. The splenic infarction of individuals affected by SCT is a benign condition that very rarely requires splenectomy and usually results in complete recovery with conservative therapies (rest, hydration, oxygen support, analgesics, and rapid patient transport to low altitudes).

Importantly, splenic infarction is not observed in SCT individuals who are native to or have been resident for many years in high-altitude locations; therefore, acclimation has a protective effect on sickling and its complications [39,40,41].

When individuals with SCT undertake sports activities of any kind at moderate altitudes or mountaineering at high altitudes, a specific acclimation program is recommended.

(B) High-pressure activities

There are no reports of complications following scuba diving. Regarding the physiology of diving (increased oxygen concentrations and possible toxicity), a minimum increase in risk can be supposed during nonprofessional practice. However, in relation to the complexity of the exercises undertaken in the navy, diving is not recommended for individuals with SCT [42].

## 4. SCT and Kidney Disease

### 4.1. Is There an Increased Risk of Developing Kidney Neoplasms in the SCT Population?

Renal medullary cell carcinoma is an exceptionally rare neoplasm that is almost exclusively described in patients with SCT, although the reasons for its prevalence are unclear.

In most cases, these patients are younger than 40; their average age is 22, and the prevalence is higher in males (3:1) aged up to 24 years. After this age, there is no gender-based difference [43,44]. The prognosis is extremely serious (mortality > 95%), with most cases presenting with metastatic disease at diagnosis [45]. The most frequent and earliest clinical manifestation is hematuria.

It is recommended that SCT carriers are informed of the potential, albeit remote, risk of renal cell carcinoma and that renal and urinary tract echotomography and, if necessary, computed tomography (CT) with a contrast agent are performed in the event of hematuria to rule out the presence of a neoplasm.

### 4.2. Is There a Greater Incidence of Renal Abnormalities in the SCT Population?

Non-neoplastic microhematuria and macrohematuria are more frequently reported in patients with SCT than in the general population [46], and older patients also have impaired urine concentrations (i.e., hyposthenuria) [46,47]. The severity of hyposthenuria is heterogeneous and depends on the quantity (%) of HbS (a detrimental factor) and on the presence of alpha-thalassemia (a protective factor) [39].

An increased incidence of pyelonephritis during pregnancy has been shown, although these conclusions are preliminary, given the limited number of patients studied [11,48,49].

### 4.3. Is There an Increased Risk of Developing Papillary Necrosis in the SCT Population?

Papillary necrosis was observed in up to 50% of patients with SCT [50], who were evaluated for the presence of symptoms, usually during in-depth clinical examinations for hematuria [51,52].

The initial presentation is mainly observed in those aged between 30 and 40 years, but the first diagnosis can also be made in older patients.

### 4.4. Is There an Increased Risk of Chronic Kidney Disease in the SCT Population?

In the past, SCT was considered a very rare cause of renal failure requiring dialysis, but the currently available data show that patients with SCT have an increased risk of chronic kidney disease (CKD) with a risk of progression leading to dialysis replacement treatment.

Adult patients affected by SCT and polycystic disease experience a more rapid progression of renal failure leading to dialysis than members of the same family with the same polycystic disease but no SCT.

In 2010, an initial study noted that the incidence of SCT in the dialyzed African American population was twice as high as that in the nondialyzed African American population [53].

Another analysis used data from five large prospective studies of African American patients. The conclusions showed an odds ratio of developing incident chronic kidney disease of 1.76, an odds ratio of having a decrease in kidney function of 1.32, and an odds ratio of having albuminuria of 1.86 for patients with SCT compared to patients without SCT [54,55,56].

Little information is available concerning the role of APOL1 variants in the SCT population. In patients with SCD and African heritage, the homozygous and compound heterozygous inheritance of APOL1 G1 and G2 variants are associated with an increased risk of proteinuria, higher albuminuria, lower eGFR, higher CKD stage, faster CKD progression, and increased risk of kidney failure [57,58,59,60]. However, few data are specific to the sickle cell trait population. In a study performed on an El Salvadoran population affected by end-stage renal disease, neither APOL1 genotype frequencies nor risk haplotype frequencies were found to be significantly associated with HbAS [61]. In another study on African Americans, no associations between the variants of APOL1 and HbAS were observed in the development of CKD [55]. In contrast with these results, in a large sickle cell trait Congolese-population-based study, high risk of APOL1 was associated with lower eGFR [62].

In view of the increased risk for SCT of developing chronic kidney disease in adulthood and due to the possibility that signs of kidney damage, such as hyposthenuria and hyperfiltration, may appear early (as early as 10 years of age), a periodic (i.e., annual) check of pediatric SCT carriers is recommended to monitor the main parameters relating to renal function (creatinine, urinalysis, and blood pressure) in order to detect kidney damage early and to delay its evolution.

### 4.5. Is There an Increased Risk of Acute Kidney Injury (AKI) in the SCT Population?

The association between SCT and AKI is not clear. Some reports describe how AKI is often associated with rhabdomyolysis in individuals with SCT [60]. A study on African American soldiers in the US reported a higher risk of AKI in individuals with SCT than in those without SCT (odds ratio (OR): 1.74; 95% CI 1.17–2.59) [63]. More recently, in 2021, a 1.6-times higher risk of severe AKI (creatinine ≥ 1.5 times above the baseline for ≥72 h) was found in African American individuals with SCT than in subjects without SCT [64].

## 5. SCT and Stroke

### Is There an Increased Risk of Stroke in Individuals with SCT?

The risk of stroke in individuals with SCT does not seem to be higher than in subjects with homozygous adult Hb. Individuals with SCT and stroke, however, have worse 30-day mortality and outcomes than patients with normal adult Hb [65,66,67].

## 6. SCT and Thrombophilia

### Is There an Increased Risk of Thrombotic and/or Thromboembolic Events in Individuals with SCT?

SCT can be considered a mild risk factor for venous thromboembolism.

In a prospective study on the African American population, it was observed that SCT was associated with a 1.5-fold increased risk of thromboembolism (VTE) compared to subjects without any HbS allele; this is mainly due to the fact of a two-fold increased risk of pulmonary embolism [68,69].

At present, no recommendation can be made regarding routine screening for thrombophilia for individuals with SCT. Further studies are needed to determine whether SCT is a risk factor for recurrent venous thromboembolism and whether there is an interaction with other risk factors, including pregnancy and the use of contraceptive hormone therapy.

Until the relevant data are available, it seems prudent to consider SCT a weak risk factor for VTE; therefore, the recommendations are similar to those given in other situations, such as the G20210A F2 mutation [70,71,72,73,74,75,76].

## 7. SCT and Bone Disease

### 7.1. Is There an Increased Risk of Hypovitaminosis D in Individuals with SCT?

Hypovitaminosis D occurs frequently in patients affected by SCD, in particular those who are of African origin; it is observed in 33–78% of children and in 60–100% of adults [77,78,79,80,81].

In the literature, no specific data are reported on the incidence of hypovitaminosis D in the SCT population.

We recommend a 25-hydroxy-vitamin D dosage at annual check-ups in both the pediatric and adult populations.

### 7.2. Is There an Increased Risk of Avascular Necrosis of the Femoral Head in Individuals with SCT?

Bone tissue involvement is very common in SCD and is responsible for chronic pain, fractures, and long-term disability [82]. Avascular necrosis is one of the most common bone complications; at the femoral level, it affects approximately one-third of patients with SCD [83].

In patients with SCT, osteonecrosis is less frequent than in individuals with SCD, but its incidence is higher than in the general population. It usually appears as avascular necrosis of the femoral head; diaphyseal infarctions are less common. A higher incidence of complications in the orthopedic surgery of these patients is not reported in the literature. Few data are available in the literature; the existing data are mainly included in case reports. The largest study consists of 29 cases of femoral head osteonecrosis associated with SCT [84,85,86,87,88,89,90,91,92,93,94,95,96,97].

The careful and meticulous monitoring of the patient is recommended during the intra- and postoperative periods, focusing on factors including saturation, hydration, acid–base balance, and temperature.

### 7.3. Is There an Increased Risk of Osteoporosis in Individuals with SCT?

No data relevant to this question have been published in the literature.

## 8. SCT and Transplants/Donation

### 8.1. Can Individuals with SCT Donate Blood or Blood Products? Can the Self-Donation of Blood or Intraoperative Blood Collection Be Performed in Individuals with SCT?

There are no studies contraindicating the donation of blood or blood components by subjects with SCT in the literature. However, some works refer to blood self-donation and intraoperative collection in which it is underlined that the autologous blood preservation procedure and any manipulations, such as washing, appear to be riskier than the collection procedure as potential causes of sickling (15–20% of sickled cells). It is, therefore, necessary to evaluate the risks and benefits on a case-by-case basis if there is a need to resort to these procedures [98,99,100,101,102].

### 8.2. Can Individuals with SCT Donate Bone Marrow Hematopoietic Stem Cells (HSCs)?

According to the Agreement, pursuant to Annex I, point 3 of Legislative decree no. 16 of 2010 between the Government, the Regions, and the Autonomous Provinces of Trento and Bolzano in the document containing “Criteria for the selection of the hematopoietic stem cell donor” [103], individuals with SCT are accepted as familiar donors if they are asymptomatic and in good condition; this is limited to the donation of bone marrow HSCs, with appropriate preoperative preparation as recommended in cases of major surgery. Predeposit for autotransfusion is not allowed.

The recommendations of the WMDA [104] (World Marrow Donor Association) allow for the possibility that a subject with SCT can donate bone marrow hematopoietic stem cells as long as the Hb values fall within the normal range.

No recent studies on individuals with SCT who have donated bone marrow HSCs are available in the literature. A study from 1961 reported that recipients of hematopoietic stem cells from donors with SCT showed no undesirable post-transplant side effects.

### 8.3. Can Individuals with SCT Donate Peripheral Hematopoietic Stem Cells (HSCs)? Is There an Additional Risk for the Donor with SCT When Donating Bone Marrow or Peripheral HSCs?

According to the Agreement, pursuant to Annex I, point 3 of Legislative decree no. 16 of 2010 between the Government, the Regions, and the Autonomous Provinces of Trento and Bolzano in the document containing “Criteria for the selection of the hematopoietic stem cell donor” [103], individuals with SCT cannot donate peripheral HSCs. They are accepted as familiar donors if they are asymptomatic and in good condition, but are limited to the donation of bone marrow HSCs.

The WMDA advises against the donation of peripheral blood stem cells by individuals with SCT.

The data available in the literature on this topic show that the mobilization of peripheral stem cells through the use of granulocyte colony-stimulating factor (G-CSF) is generally safe and well tolerated in individuals with STC. Adverse events related to mobilization do not differ from those that occur in subjects without SCT, and no sickle cell crises are reported. However, it has been noted that the incidence of these adverse events seems to be more frequent [105,106,107,108,109,110,111]. Some works report that mobilization with G-CSF, apheresis treatment of large volumes of blood, manipulation, and cryopreservation were found to be safe in patients with SCT [105]. Further studies report that the African American population shows a significantly better response to G-CSF than the Caucasian population in terms of the mobilization of CD34+, independent of other demographic and hematological parameters. Adverse events and the mobilization of CD 34+ were found to be similar in African American donors with and without SCT [112].

### 8.4. Can Individuals with SCT Donate Organs?

Few cases are reported in the literature of organ transplants from a living or deceased donor with SCT, although an underestimation of these records is possible since the search for HbS is not part of the organ donor screening. However, based on the literature, it is not possible to exclude subjects with SCT as donors a priori.

From the analysis of the literature, with regard to living donors, it seems that the presence of sickle cell disease in the donor is to be considered an absolute contraindication due to the greater incidence of kidney dysfunction and of other related comorbidities that can put both the donor and the possible recipient at risk. Meanwhile, in the case of SCT in the absence of renal alterations, it is possible to consider these subjects as potential donors. The lack of “policy and practice” in most transplant centers and of national and international guidelines for the management of such donors determines practices that are not always unequivocal in the cases of living donors with SCT [113,114].

As far as kidney transplantation from a deceased donor with SCT is concerned [115], there are very few cases of kidney transplant reported in the literature, but SCT should not be considered an absolute contraindication, as long as a morphological and functional evaluation of the organ is carried out.

Considering these limitations and the doubts connected to them, most transplant centers accept patients with SCT as donors if the disease has never appeared clinically [114]. 

Given the shortage of organs available for transplantation, deceased donors with sickle cell disease [116] should not be excluded a priori from kidney donation. Moreover, in the absence of renal dysfunction, with normal tissue architecture evaluated by a pretransplantation biopsy and in the absence of other disease-correlated comorbidities, it is possible to consider these individuals as potential donors, upon a risk–benefit assessment of the recipient.

Regarding the donation of other organs, once their suitability from a morphological and functional point of view and the absence of other comorbidities have been confirmed, there are no reasons to justify their non-use.

### 8.5. Does Organ Donation Pose an Additional Risk to Donors with SCT?

Given the shortage of data reported in the literature on kidney transplantation from living donors with SCT, it is difficult to evaluate additional risks compared to the normal population, even though no serious adverse events have been reported [117]. The kidney function of potential donors must still undergo a careful assessment before and after nephrectomy and, during nephrectomy, the instructions for major surgeries must be followed.

### 8.6. Does Organ Transplantation Pose an Additional Risk to Individuals with SCT?

The literature contains case reports showing some complications, such as the sickling of the transplanted kidney after the transplant and the onset of perioperative focal cortical necrosis in recipients with SCT [118,119]. Nevertheless, from the viewpoint of the patient and from the perspective of transplant survival, kidney recipients who have sickle cell disease or SCT show post-transplant outcomes comparable to those of the general population, even if the onset of sickle cell crises is possible, especially in patients with SCD and during the first year after surgery [120].

The possible causes of sickling in renal transplantation include [121,122,123,124,125,126] anesthetic drugs, which have the potential to worsen the hypoxic state of the patients and can precipitate vaso-occlusive crises intra- or immediately postoperatively; ischemia/reperfusion injury in the early post-transplantation period; dehydration resulting from fluid deprivation, excessive fluid loss, and an inability to ingest fluids; improved erythropoiesis resulting from the restoration of erythropoietin levels by the kidney transplant; infections related to the patient’s immunosuppressive status; and tissue ischemic effects mediated by calcineurin inhibitor drugs (cyclosporine and tacrolimus).

An unknown SCT condition may be a risk factor for the early rejection of a transplanted kidney; therefore, it is important to evaluate SCT during the pretransplant screening of the recipient [127].

In addition to kidney transplantation, case reports of patients undergoing other types of organ or tissue transplants are reported in the literature. Some of these reports mention important complications, such as that relating to the death of a female patient after a heart transplant [128], whereas others report complications that resolved spontaneously (e.g., a spontaneously resolved post-heart-transplant priapism).

Some case reports of tissue transplants are also reported, such as a mastoplasty from autologous tissue without complications [129].

However, to avoid intra- or postoperative complications in the event that an individual with SCT must undergo a transplant procedure, especially an organ transplant, the instructions for major surgeries must be followed [130].

### 8.7. Can Subjects with SCT Receive Blood, HSCs, or Organs from Subjects with SCT?

No data relevant to this question are reported in the literature, with the exception of a case of kidney transplantation in a patient with SCT from a donor with SCT [115]. In this case, no complications were reported for either patient both immediately post-transplant and after four years of follow-up.

## 9. SCT and Neonatal/Pediatric Age

### Is There an Increased Risk of Growth Delay in Children with SCT?

Little information is available on the growth and nutrition of children with SCT. An English study (a comparison of three groups of young people at a college, 50 without SCT, 50 with SCT, and 50 with sickle cell disease) showed no differences in the growth, body mass, and physical performance between those with and without SCT [131]. An Iraqi study showed a high incidence of iron deficiency (approx. 70%), especially in children aged < 5 years [132].

In the absence of definite data, an annual clinical check-up of SCT carriers during growth is advised, with evaluation of iron, folic acid, and vitamin D supplies.

## 10. SCT and Pregnancy

Few studies discuss the impact of SCT on pregnant women in relation to the preconceptional phase, pregnancy, and the postpartum period; the existing studies have limitations related to their retrospective nature and the size of the sample studied. Burgos Luna et al. highlight that SCT status is under-diagnosed in pregnant women, which strongly influences the collection of accurate information regarding the related complications [133].

We recommend testing for hemoglobinopathies (HbS and thalassemia) for all women considering pregnancy and/or pregnant women with anemia, microcytosis, or a family history of hemoglobinopathies, or those from regions endemic for sickle cell disease and thalassemia.

If the woman is a carrier of beta-thalassemia or HbS, the partner should be tested for HbS and thalassemia, and specialist advice should be provided.

Furthermore, it is considered necessary to rule out the presence of the sickle cell trait in pregnant women with anemia and severe osteoarticular pain that is poorly responsive to analgesic therapy [133,134].

### 10.1. Is There an Increased Risk of Anemia in Pregnant Women with SCT?

Pregnant SCT patients generally have more pronounced anemia than healthy women of the same age and ethnicity. Despite the small number of related studies, folate deficiency is generally recognized as an important factor of anemization in pregnant women with SCT, often in conjunction with iron deficiency.

Although there are no relevant randomized studies, folic acid supplementation is recommended throughout pregnancy and breastfeeding, as well as iron supplementation if a status of iron deficiency is identified on the basis of ferritin and transferrin saturation levels [133,135,136,137].

### 10.2. Is There an Increased Risk of Preeclampsia–Eclampsia in Pregnant Women with SCT?

Data on the risk of pregnancy-related hypertensive disease, preeclampsia, and eclampsia in pregnant women with SCT are not conclusive. These complications represent the main cause of maternal mortality in Western countries. In women with SCT, the risk of pregnancy-related hypertensive disease is increased, while there seems to be no clear association between SCT and preeclampsia/eclampsia [48,136,138].

Close monitoring of blood pressure and proteinuria in patients with SCT is recommended during pregnancy.

### 10.3. Is There an Increased Risk of Urinary Tract Infections in Women with SCT during Pregnancy?

In women with SCT, an increased susceptibility to acute cystitis and asymptomatic bacteriuria and a higher risk of pyelonephritis during pregnancy have been described compared to the control population. In a subsequent study, Tita et al. did not confirm this observation in a larger population [139].

In the absence of conclusive data, we deem it appropriate to proceed according to a principle of caution, recommending serial controls with urine cultures during pregnancy and early and intensive treatment of pregnant women with SCT whose clinical picture is suggestive of pyelonephritis [48,49,139].

### 10.4. Is There an Increased Risk of Thrombotic Complications in Women with SCT during Pregnancy and in the Postpartum Period?

Individuals with SCT have an increased risk of thrombotic complications, such as deep venous thrombosis or pulmonary embolism, compared to the healthy population. Pregnancy itself is a condition associated with an increased thrombotic risk. At present, there are no conclusive results regarding the thrombotic risk for pregnant women with SCT, even if some studies have identified a greater risk of deep venous thrombosis in pregnant SCT women.

A careful anamnestic reconstruction of the patient’s history is recommended, in particular with regard to previous thrombotic events and/or miscarriages, as well as a close follow-up examination during pregnancy.

### 10.5. Is There an Increased Risk of Miscarriage in Pregnant Women with SCT?

There are no studies designed to evaluate the risk of miscarriage in women with sickle cell trait; therefore, the available data are conflicting and nonconclusive [48,136,139].

### 10.6. Is There an Increased Risk of Preterm Delivery in Pregnant Women with SCT?

The data available to us show an increased risk of preterm delivery in women with SCT. These results are important but should be considered preliminary [140] because of the sample size.

### 10.7. Is There an Increased Risk of Fetal Growth Delay and/or Low Birth Weight in Pregnant Women with SCT?

Studies do not suggest that sickle cell trait has an effect on fetal growth or on the weight of newborns at birth. A US study shows an increased risk of fetal growth delay in pregnant African American women compared to the control population of the same age and of different ethnicities [48,141,142].

### 10.8. Is There an Increased Risk of Fetal or Perinatal Morbidity and/or Mortality in SCT?

Sickle cell trait has no clear effect on fetal or perinatal morbidity and/or mortality. A recent retrospective, cross-sectional, case-control study carried out in Bahrain highlights an increased risk of intrauterine fetal death in pregnant Bahraini women compared to the control population without SCT [143].

### 10.9. Is There Increased Maternal Morbidity and/or Mortality in Women with SCT?

There is no evidence of the sickle cell trait affecting maternal mortality during postpartum hospitalization. There is an increased risk of re-hospitalization for major or minor postpartum complications for women with SCT at 30 and 90 days after discharge.

Multidisciplinary follow-up examinations and multidisciplinary evaluation are recommended, with the involvement of the disease specialist, in the weeks after delivery [144].

### 10.10. Is There an Increased Risk of Thrombotic Complications in Women with SCT Who Are on Estroprogestin Therapy?

Patients with SCT have an increased risk of thrombotic complications, such as deep venous thrombosis or pulmonary embolism, compared to the healthy population. The data concerning women with SCT on estroprogestin therapy are not conclusive but indicate a possible synergic effect in a pro-thrombotic sense with SCT [48,145].

## 11. SCT and Ocular Alterations

### Is There an Increased Risk of Ocular Complications in Individuals with SCT?

Proliferative retinopathy is present in 20% of adult patients affected by SCD, usually between the fourth and the fifth decade of life [146]. There is no evidence of higher incidence of this complication in subjects with SCT. Isolated cases of proliferative retinopathy have been reported in SCT individuals; in most cases, it coexists with diabetes mellitus or other comorbidities that could explain the retinopathy [147,148].

Under the conditions of severe dehydration and/or hypoxia as a result of traumatic events or eye surgeries, individuals with SCT are more susceptible to developing complications, such as infarct phenomena that affect the retinal artery and optic nerve, glaucoma, hemorrhage, and optic nerve atrophy [146].

SCT individuals must be assessed early in the event of ocular trauma, as complications are often disproportionate to the size of the hyphema [147].

In the event of eye surgery, adequate preparation by transfusion prophylaxis should be considered (simple transfusion or transfusion with manual or automated red cell exchange) [130].

## 12. Miscellanea

### 12.1. Is There an Increased Risk of Splenic Infarction in Individuals with SCT?

Individuals with SCT may develop splenic infarctions under the conditions of low oxygen tension and high altitudes, such as flights in unpressurized cabins or exercise in the mountains [39,148]; however, they might also occur under rest conditions at high altitudes or when exercising at sea level [149].

Usually these are minor, paucisymptomatic, and self-limiting events, sometimes diagnosed during imaging tests, such as ultrasounds or abdominal CT scans, that are performed for other reasons. Cases of acute splenic infarction with anemization have occasionally been described.

However, in the most serious cases, splenic rupture may occur, necessitating an emergency splenectomy [41].

Splenic infarction is not observed in SCT individuals who are born in or reside for years in high-altitude locations; therefore, acclimation has a protective effect on sickling and its complications.

The possibility of acute splenic sequestration should be considered in individuals whose SCT defect coexists with spherocytosis [41].

### 12.2. Should Individuals with SCT Be Vaccinated like Patients with Sickle Cell Disease?

Children with SCT seem to have a higher incidence of pneumococcal infections and serious events [150]. The complete vaccine panel, including antipneumococcal vaccination with both conjugate and polysaccharide vaccines, is indicated in individuals with SCT.

In the presence of documented splenic infarctions, we deem it appropriate to consider the subjects to be just like patients with SCD (that is, with functional hyposplenism/asplenia) and, therefore, to proceed with the required vaccinations (SITE Recommendations, Asplenia Algorithm [151]).

### 12.3. Do Individuals with SCT Have Higher Morbidity and/or Mortality Related to SARS-CoV2 Infection?

Based on the literature review, it appears that most COVID-19 patients with SCT do not differ from the general COVID-19-infected population in terms of the clinical course of the infection, the hospitalization rate, and infection-related mortality. Therefore, unlike sickle cell syndrome, which is a known risk factor for COVID-19-related complications, the risk for SCT is not established [152].

### 12.4. Can a Subject with SCT Develop Vaso-Occlusive Crises (VOCs)? Are There Risk Factors for VOCs in Subjects with SCT?

Vaso-occlusive crises (VOCs), acute chest syndrome (ACS), and acute anemization are also described in SCT individuals, albeit rarely [11,153,154,155,156]; they are mainly associated with predisposing extreme conditions (high altitude, sustained or extreme exercise, dehydration, low oxygen tension, and increased pressures, such as those experienced during scuba diving).

In most of the cases described, SCT was, in fact, associated either with the coexistence of other Hb mutations (for example, HbS-Antilles; Hb-Quebec-Chori; HbS-Oman; Hb-Jamaica) or with other red blood cell defects (spherocytosis, pyruvate kinase deficiency, etc.). It is important to note that those who inherit another mutation of beta-hemoglobin (for example: beta-thalassemia, Hb C) should be considered as having sickle cell syndrome, albeit in forms with variable severity compared to homozygosity SS.

### 12.5. Is There an Increased Risk of Adverse Events or Complications in Individuals with SCT during Ramadan Fasting?

Prolonged fasting with the abstention from liquids that is also observed during the Ramadan month can induce a state of severe bodily dehydration.

The increased viscosity that is secondary to dehydration can be particularly dangerous in individuals with SCT [157] in relation to kidney complications following the renal medulla’s exposure to a hypertonic environment.

While respecting traditions and religious belief, it is of fundamental importance that specialist doctors inform patients of the risk of increased viscosity and vaso-occlusive crises that might occur during the fasting period in individuals with SCT who, for reasons related to their jobs or participation in sporting activities, are more exposed to the risk of dehydration, with the consequent need to implement all relevant preventive measures to safeguard their health.

### 12.6. Is There an Increased Risk of Adverse Events or Complications in Individuals with SCT Who Undergo Surgery?

In the literature, there are isolated reports of adverse events in SCT patients following surgical interventions due to the fact of hypoxia or reduced perfusion during surgery. Some authors recommend erythrocyte exchanges to reduce the percentage of HbS before some major surgical procedures, such as cardiac surgery for cardiopulmonary bypass [158], or transplant or thoracic surgery. In fact, during cardiopulmonary bypass procedures, many of the factors that favor sickling, such as stress, hypothermia, dehydration, hypoxia, inflammation, acidosis, and infections, can occur [159]. In the literature, many case reports/series describe the use of erythrocyte exchanges in the pre- and postsurgical periods with the aim of reducing HbS to levels below 30%, thus decreasing the risk of sickling and vaso-occlusive events [159,160,161,162]; however, many case reports/series also describe favorable outcomes in patients with SCT who underwent cardiac surgery with HbS values > 30% and who were not prepared with erythrocyte exchanges in the pre- and postsurgical periods [163,164,165].

A controlled study demonstrated that there are no additional risks due to the fact of surgical interventions in nontransfused individuals with SCT (as compared to individuals with normal Hb), including cases of intrathoracic surgery [166]. Another controlled study of open heart surgery in Africa showed no adverse events related to sickling in 11 patients with SCT and in 2 with double heterozygosity [167]. However, two of the patients with SCT died as a result of surgical complications; the authors attributed these deaths to the patients’ severe heart injuries rather than to the consequences of sickling.

We recommend considering prophylactic transfusional therapy before eye surgeries or high-risk surgeries in order to decrease the risk of adverse events related to sickling [130]. The transfusional procedure should be performed within a week of the scheduled surgery. Erythrocytapheresis (automated red cell exchange or manual exchange) may be used to bring HbS values to percentages < 30%, or simple transfusions may be performed in cases of anemia. Surgical teams and anesthetists should be aware of the possibility of adverse events in SCT patients following surgical interventions due to the fact of hypoxia or reduced perfusion.

## 13. Conclusions

Sickle cell trait is one of the most common hemoglobin carrier states in the world. Although it usually affords carriers a normal life expectancy and normal quality of life, it can be associated with some complications of sickle cell disease, albeit very rarely and under certain metabolic or environmental conditions, or in conjunction with red blood cell defects. Therefore, it is essential to identify patients’ SCT carrier status, above all because of the positive consequences that this information might have both for the awareness of the reproductive risk and for the health of the subject. Moreover, increasing knowledge about these clinical manifestations and their prevention and management can be a useful tool for all healthcare providers involved in this issue.

## Figures and Tables

**Table 1:** Major clinical complications of sickle cell trait and proposed counseling recommendations.

Complication	Counseling Recommendations
Renal medullary carcinoma	In the event of micro- or macroscopic hematuria, prompt referral to renal imaging (echo or abdominal contrast CT scan)
Chronic kidney disease	Early identification of kidney damage through an annual check of serum creatinine levels, chemical urinalysis, and periodic blood pressure measurement
Stroke	No evidence of increased risk of early-onset stroke with SCT; additional work-up to identify causes; 30-day mortality worse than without SCT
Venous thromboembolism	SCT is a weak risk factor; decisions regarding choice and duration of anticoagulation should not be influenced by the presence of SCT
Venous thromboembolism during pregnancy	Collecting history regarding prior thromboembolic events and/or miscarriages; close monitoring for thromboembolic events
Hypertension during pregnancy	Close monitoring for blood pressure and proteinuria
Urinary infection during pregnancy	Serial testing for chemical urinalysis and urine culture, early antibiotic treatment in case of infection or suspected pyelonephritis
Hyphema, eye trauma, and eye surgery	Monitoring for increased risk of ischemic complications; consider prophylactic transfusional therapy before surgery (simple transfusion or erythrocytapheresis)
High-risk surgery (cardiac surgery, intrathoracic surgery, brain surgery, transplant surgery, major vascular surgery, major spine surgery, surgeries requiring prolonged general anesthesia for >4 h)	Consider prophylactic transfusional therapy within a week of the scheduled surgery: erythrocytapheresis (automated red cell exchange or manual exchange) to bring HbS values to percentages of <30%, or simple transfusion in the case of anemia

CT: computed tomography; SCT: sickle cell trait.

**Table 2:** Exertional collapse associated with sickle cell trait.

Documented Sports Activities	Clinical Picture	Evolution
Football, training, cross-country racing, swimming, spinning, hockey, military exercises	Extreme muscle weakness, pain, even mild, with rapid onset without prodromes	Initially conscious subject, Loss of consciousness, Mental confusion, Tachycardia, arrhythmia, Death
**Causes of Exertional Sickling**	**Inducing Factors**	G**enetic Factors**
Extreme physical effort for as little as a few minutes (2–5), even in trained subjects/professional athletes	Prolonged intense exercise, dehydration, high temperatures, altitude, suboptimal physical conditions, fever; intake of the following drugs: antipsychotics, antihistamines, decongestants, statins; intake of caffeine-stimulant drinks	Protective: high levels of fetal Hb (HbF); coexistence of alpha-thalassemia Aggravating; high amount (%) of HbS; coexistence of red blood cell membrane or enzymatic defect

## Data Availability

Not applicable.
